# AI for medical use

**DOI:** 10.18632/oncotarget.26556

**Published:** 2019-01-04

**Authors:** Masamitsu Konno, Hideshi Ishii

**Affiliations:** Department of Medical Data Science, Graduate School of Medicine, Osaka University, Suita, Osaka, Japan

**Keywords:** artificial intelligence, convolutional neural network, diagnosis, surgery, medicine

**Figure 1:**
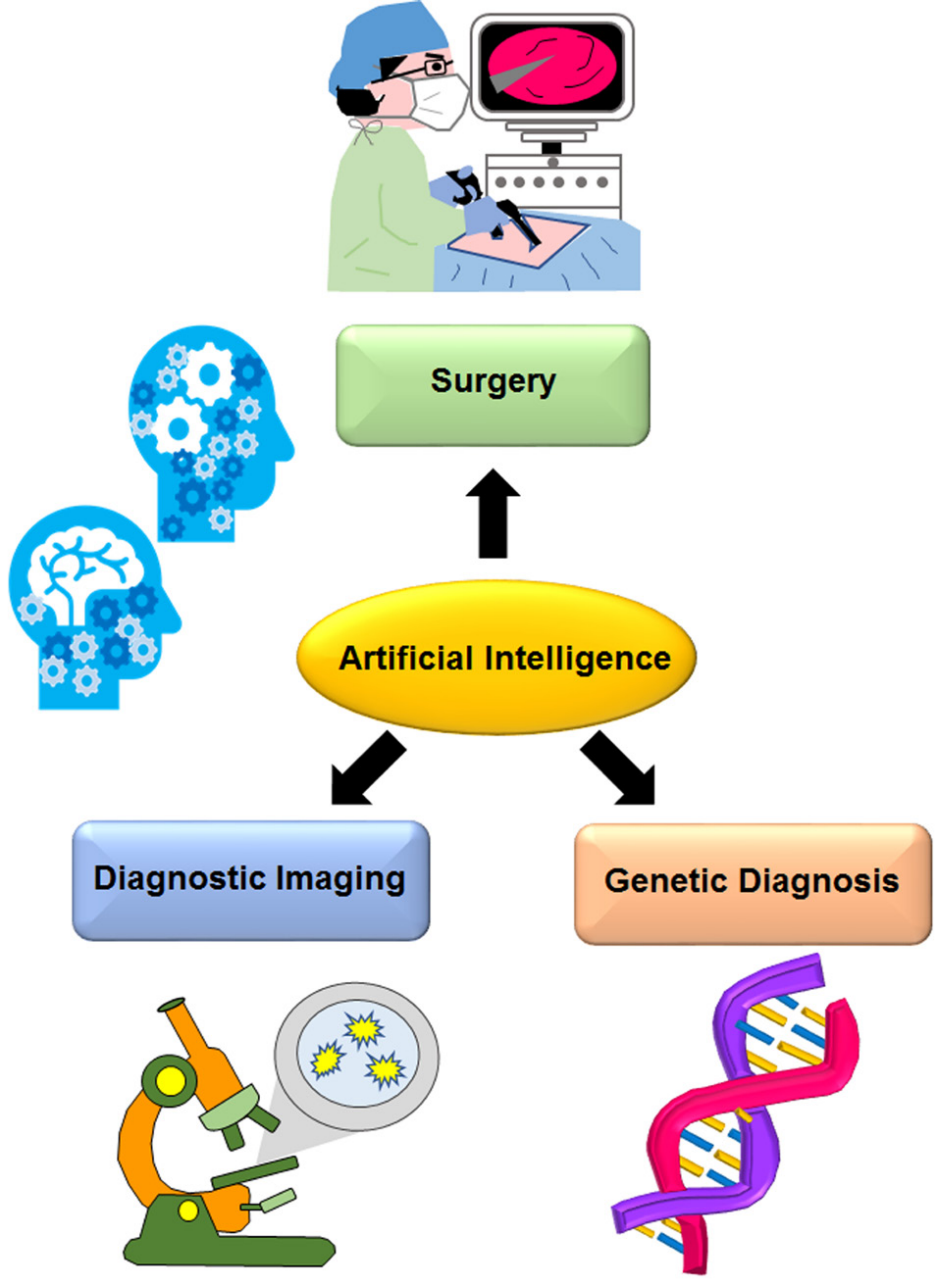
Artificial intelligence for clinical use It has been recently reported that artificial intelligence has a potential to be used in the diverse fields of diagnostic imaging, genetic diagnosis, surgery, or other medical applications.

In the field of artificial intelligence (AI), developments are leading to a new era involving its social implementation. In 2006, Hinton et. al. reported that high-dimensional data can be converted into low-dimensional codes by training a multilayer neural network with a small perceptron [1]. This discovery has triggered developments in AI. In recent years, the technology involving AI trained with a convolutional neural network (CNN), which mimics the optical nerve network, has been developed. Various attempts have been made to train AI with medical images for its utilization in clinical applications (Figure 1). Esteva et. al. published the first report on clinical AI [2], wherein they trained a CNN using 129,450 clinical images, which consisted of 2,032 different diseases. Surprisingly, the performance of the CNN was comparable with the level of competency shown by dermatologists in classifying skin cancer. We recently trained a CNN using10,000 images each of radiosensitive and radioresistant cancer cells [3]. The accuracy of this model was very high (96%). Features extracted by the CNN were plotted using t-distributed stochastic neighbor embedding, and it was confirmed that each cell line was well clustered. Rajpurkar et. al. reported the results on X-ray image diagnosis [4]. They established a novel algorithm, CheXNet, which comprised a 121-layer CNN. This CheXNet network was trained using the ChestX-ray14 dataset, which contained 112,120 frontal-view chest X-ray images that were individually labeled with 14 different thoracic diseases. Four practicing academic radiologists annotated a test set, and the performance of CheXNet was compared to that of the radiologists. The performance of the CheXNet algorithm was found to exceed the average radiologist performance. Another group developed AI for the diagnosis of cancer metastasis [5]. Liu et. al. made a CNN to automatically detect and localize tumors as small as 100 × 100 pixels in a large-sized image (100,000 × 100,000 pixels). This CNN was trained using the Camelyon16 dataset, which included hematoxylin and eosin-stained whole-slide images of cancer metastasized lymph node sections. This network could detect 92.4% of the tumors, relative to the 82.7% detected by the previous optimal automated approach. At the same time, a pathologist performing an exhaustive search achieved 73.2% sensitivity. This AI achieved image level AUC scores > 97% on the Camelyon16 test set and an independent set of 110 clinical sample slides. AI has also been used for the detection of vascular diseases [6]. Santini et. al. made a system for the automatic quantification of calcium scores in ECG-triggered non-contrast-enhanced cardiac computed tomography images. This system used a CNN for the segmentation and classification of candidate lesions as coronary or not, previously extracted in the region of the heart using a cardiac atlas. This network was trained with 45 CT volumes. Individual lesions were detected with a sensitivity of 91.24%, specificity of 95.37%, and positive predicted value of 90.5%. After comparing calcium scores obtained by the system and those manually scored by an expert operator, a Pearson coefficient of 0.983 was obtained. AI has been used not only for diagnostic imaging but also for medical records [7]. Nguyen et. al. developed Deepr (short for Deep record) a novel end-to-end deep learning system that learns to extract features from medical records and automatically predicts future risk. Deepr transforms a clinical record into a sequence of discrete elements separated by coded time gaps and hospital transfers. Deepr achieved superior accuracy compared with traditional techniques. It is able to detect meaningful clinical motifs from medical records, and these predictions have been applied to AI [8]. Bajor et. al. also analyzed clinical records using a neural network. They used a recurrent neural network, which was suitable for analyzing temporal sequence data. Their model achieved high prediction accuracy (micro-averaged AUC, 0.93; label ranking loss; 0.076), limited by hardware constraints on the model size. AI can also be used for gene diagnosis [9] to integrate multi-omics data from high-risk neuroblastoma. Zhang et. al. adopted the autoencoder, and combined it with K-means clustering to identify two neuroblastoma subtypes with significant survival differences. They also validated the classification in two independent datasets by training machine-learning classification models, and confirmed its robustness. This analysis showed that MYCN amplification more frequently occurred in the ultra-high-risk subtype, in accordance with the overexpression of MYC/MYCN targets in this subtype. Of note, AI has been started to be applied in surgery [10]. Shademan et. al. created a computer program to generate a plan to complete complex surgical tasks on deformable soft tissue, such as suturing and intestinal anastomosis. They compared metrics of anastomosis, including the consistency of suturing informed by the average suture spacing, the pressure at which the anastomosis leaked, the number of mistakes that required removal of the needle from the tissue, completion time, and lumen reduction in intestinal anastomoses. This system could demonstrate that the outcome of supervised autonomous procedures was superior to surgery performed by expert surgeons. Although these reports have not demonstrated actual clinical use, they indicate that the potential of medical treatment using AI will be recognized in the near future.
